# Prevalence of feet and ankle arthritis and their impact on clinical indices in patients with rheumatoid arthritis: a cross-sectional study

**DOI:** 10.1186/s12891-019-2773-z

**Published:** 2019-09-11

**Authors:** Sung Won Lee, Seong-Yong Kim, Sung Hae Chang

**Affiliations:** 1 Division of Rheumatology, Department of Internal Medicine, Soonchunhyang University Cheonan Hospital, Cheonan, Korea; 2 Division of Rheumatology, Department of Internal Medicine, Soonchunhyang University College of Medicine, Cheonan, Chungchungnam-do Korea; 3 Division of Big Data and Management Engineering, Hoseo University, Asan, Korea

**Keywords:** Rheumatoid arthritis, Foot, Ankle, Disease activity index, Biologics therapy, Metatarsophalangeal joints

## Abstract

**Background:**

We aimed to evaluate the prevalence of foot and/or ankle arthritis (FAA) and its impact on clinical indices in patients with rheumatoid arthritis (RA).

**Methods:**

This cross-sectional study used data from the Korean College of Rheumatology Biologics & Targeted therapy registry to observe clinical outcomes of patients undergoing biologics therapy and conventional therapy. FAA was defined as ≥1 tender or swollen joint in the ankle and/or 1st-5th metatarsophalangeal (MTP) joints. Disease Activity Score 28 (DAS28), Routine Assessment of Patient Index Data 3 (RAPID3), Simplified Disease Activity Index (SDAI), and Clinical Disease Activity Index (CDAI) were assessed.

**Results:**

Among 2046 patients, 598 had FAA. The ankle joint was the most commonly involved joint in FAA (tender joint, 71.4%; swollen joint, 59.5%), followed by the third and second MTP joints. Patients with FAA showed higher DAS28, RAPID3, SDAI, and CDAI scores. FAA presence was significantly associated with non-remission as per DAS28-ESR (odds ratio, 3.4; 95% confidence interval, 2.0–5.8), DAS28-CRP (3.6, 2.4–5.3), SDAI (6.3, 2.8–14.6), CDAI (7.6, 2.4–24.3), and RAPID3 (5.6, 2.7–11.5) indices on adjusting for age, sex, disease duration, presence of rheumatoid factor, presence of anti-cyclic citrullinated peptide antibody, lung disease, use of methotrexate, and previous use of biological disease-modifying anti-rheumatic drugs. Patients with FAA were less likely to achieve remission of SDAI (*n* = 6, 1.0%) and CDAI (*n* = 3, 0.5%) than that of DAS28-ESR (*n* = 21, 3.5%), DAS28-CRP (*n* = 38, 6.4%), and RAPID3 (*n* = 12, 2.0%).

**Conclusions:**

FAA represents a severe disease activity and is an independent risk factor for non-remission in patients with RA.

**Electronic supplementary material:**

The online version of this article (10.1186/s12891-019-2773-z) contains supplementary material, which is available to authorized users.

## Background

Rheumatoid arthritis (RA) is a systemic inflammatory disease that can involve the synovial joint. Foot and ankle are commonly affected, and more than 90% of patients with RA reported foot pain during the course of their disease [1]. Even in patients with early RA, 70% of patients had foot synovitis less than 3 years since the onset of symptoms. Radiographic damage to the feet was observed in about one-fifth of the patients, and the proportion increased to 60% after 8 years [2]. Foot arthritis results in impaired foot function, which is associated with frequent falls [3].

RA can be assessed using various parameters; the disease activity indexes are comprised of multiple factors, including patient’s global assessment (PGA), evaluator’s global assessment (EGA), C-reactive protein (CRP), or erythrocyte sedimentation rate (ESR) to measure joint inflammation and disease activity. The most extensively validated and widely used index is the Disease Activity Score (DAS). However, the formula for DAS using 28 joint counts is complicated [DAS28 = (0.56 * tender joint count 1/2) + (0.28 * swollen joint count 1/2) + (0.7 * ln [ESR]) + (0.014*VAS)] [4, 5]. Therefore, simplified disease activity index (SDAI) or clinical disease activity index (CDAI) was developed, which offers simpler calculation with the arithmetic sum of swollen joint counts (SJS), tender joint counts (TJC), PGA, EGA, CRP for SDAI and SJS, TJC, PGA, EGA for CDAI for CDAI [4, 6]. All three indices are correlated [4, 7]. However, the disease activity index excludes foot and ankle joints, and there have been controversies whether these composite indices represent the actual disease activity involving foot and ankle joints [8–11]. Wechalekar et al. reported that 43% of patients with a DAS28-ESR of < 2.6 had foot synovitis, and 25–36% of patients with remission as per SDAI and CDAI had foot synovitis [8].

In the current study, we investigated the prevalence of FAA, an association of FAA with disease activity indices, and the impact of FAA on disease activity. Although there are several studies on the prevalence or disease activity in patients with FAA, the current study is the one with the most significant number of patients.

## Methods

The Korean College of Rheumatology Biologics & Targeted therapy (KOBIO) registry, a multicenter hospital-based observational registry designed by the Korean College of Rheumatology (KCR), was established in 2012 to assess clinical courses, outcomes, and adverse events in patients on biologic disease-modifying anti-rheumatic drug (bDMARD) therapy that was explained in a previous study [12]. Patients with RA were recruited from 38 hospitals in South Korea. Briefly, all patients satisfied the 2010 ACR/EULAR RA classification criteria or 1987 ACR RA classification criteria. Baseline demographic data were collected including age, gender, body mass index (BMI), and smoking status at the time of enrolment and annually thereafter. Laboratory data, including rheumatoid factor (RF), anti-cyclic citrullinated peptide (anti-CCP) antibody, CRP, and ESR, and current medications, including the use of glucocorticoids disease-modifying anti-rheumatic drugs (DMARDs) such as methotrexate (MTX) and bDMARDs, were investigated by review of medical records. The treating physician performed 44 SJCs, 44 TJCs, and EGA. The treating physicians counted the swollen or tender joints only considered to be associated with RA. Radiographic damage, i.e., presence of joint space narrowing or bone erosion was surveyed by either the treating rheumatologist or a radiologist. PGA and Routine Assessment of Patient Index Data 3 (RAPID3) were also evaluated. DAS28-ESR, DAS28-CRP, SDAI, and CDAI were also assessed. Remission status was also evaluated using the 2011 ACR/EULAR Boolean-based remission criteria [13]. All items were assessed at enrolment and at annual follow-up visits; however, in the current study, we used only the baseline data. Written informed consent was obtained from all participants. The study was approved by the ethics committee or institutional review boards of each centre and was conducted in accordance with the Helsinki Declaration of 1975 as revised in 2008. All patient data were transferred by individual investigators to the KOBIO web server (http://www.rheum.or.kr/kobio/). In the current study, FAA was defined as one or more tender or swollen joints in the ankle and/or the first to fifth metatarsophalangeal (MTP) joints.

### Statistical analysis

Data are presented as mean ± standard deviation or percentage for patients with FAA or those without FAA, as appropriate. Means were analysed using the Student’s *t*-test for parametric variables, and the Mann-Whitney test for the non-parametric variables according to the normality of the variables. Categorical variables were compared using the chi-square or Fisher’s exact test. *P* values ≤0.05 were considered significant. Logistic multivariate regression analysis was performed to clarify if FAA was statistically significant as an independent risk factor for non-remission. Factors known to be associated with remissions, such as age, sex, duration of disease, presence of RF, presence of anti-CCP, existence of lung disease, use of MTX, and previous use of bDMARDs were included for multivariate analysis. TJC, SJC, EGA, PGA, ESR, and CRP were not included in the multivariate analysis because they were included in the dependent factors (i.e. disease activity indices). All analyses were performed using the PASW Statistics 18 (SPSS Inc., Chicago, IL, USA).

## Results

### Prevalence of FAA and clinical characteristics of patients with FAA

Of 2046 patients registered by March 2017, 598 (29.2%) had FAA. The age at enrolment was comparable between patients with FAA and those without FAA. Females had higher incidence rate of FAA [30.1% (*n* = 520 of 1729) in females vs 24.6% (*n* = 78 of 317) in males, *p* = 0.05]. Patients with FAA had a longer duration of the disease than those without. The BMI was similar between the two groups. Current or ex-smoker to non-smoker rate was comparable between the two groups. No significant difference was noted between groups with regard to anti-CCP antibody positivity rate. There were no significant intergroup differences in terms of steroid use, but the doses were significantly different between groups (5.2 ± 10.7 mg/day for patients with FAA vs 4.3 ± 4.2 mg/day for those without FAA; *p* = 0.003). The proportion of patients with previous use of bDMARDs was higher in the FAA group than that in the non-FAA group (Table 1).

**Table 1:** Demographic and clinical data of patients with and without foot and/or ankle arthritis

	With FAA	Without FAA	*P*-values
	(598, 29.2%)	(1448, 70.8%)	
Demographic Characteristics			
Female, *n* (%)	520 (87.0)	1209 (83.5)	0.05
Age at the time of enrollment, years	54.1 ± 12.6	54.4 ± 13.0	0.63
Disease duration, years	8.5 ± 8.3	7.4 ± 7.0	< 0.01
Disease duration < 1 year, *n* (%)	99 (16.6)	228 (15.8)	0.69
Disease duration < 2 years, *n* (%)	170 (28.5)	399 (27.7)	0.70
Disease duration < 3 years, *n* (%)	200 (33.6)	509 (35.3)	0.47
Disease duration < 4 years, *n* (%)	241 (40.4)	622 (43.1)	0.28
Disease duration < 5 years, *n* (%)	273 (45.8)	71 (49.6)	
Disease duration ≥5 years, *n* (%)	323 (54.2)	727 (50.4)	0.12
Body mass index, kg/m^2^	22.6 ± 3.6	22.6 ± 3.2	0.97
Current/ex-smoker, *n* (%)	89 (14.9)	217 (15.0)	1.00
Presence of RA-associated lung diseases, *n* (%)	21 (3.5)	47 (3.3)	0.79
Positive for rheumatoid factor, *n* (%)	491 (85.1)	1174 (83.5)	0.38
Positive for anti-cyclic citrullinated peptide, *n* (%)	404 (84.7)	1046 (85.1)	0.88
Radiographic damage			
Hand X-ray			
Erosion, *n* (%)	197 (40.0)	417 (34.8)	< 0.05
Joint space narrowing, *n* (%)	226 (45.8)	555 (46.5)	0.83
Feet X-ray			
Erosion, *n* (%)	153 (36.7)	247 (29.1)	< 0.01
Joint space narrowing, *n* (%)	107 (25.7)	224 (26.5)	0.79
Medication			
Current glucocorticoid use, *n* (%)	497 (83.1)	1169 (80.7)	0.21
Daily dose (prednisolone equivalent), mg	5.2 ± 10.7	4.3 ± 4.2	< 0.01
Current use of MTX, *n* (%)	567 (94.8)	1337 (92.3)	0.05
Previous use of bDMARDs, *n* (%)	145 (24.2)	233 (16.1)	< 0.01
Disease activity			
Swollen joint count (44 joints examined)	8.9 ± 6.8	3.9 ± 4.4	< 0.01
Tender joint count (44 joints examined)	11.9 ± 8.6	4.9 ± 5.0	< 0.01
Patients Global Assessment score (1–10 mm)	6.7 ± 2.3	5.7 ± 2.6	< 0.01
Evaluator’s Global Assessment score (1–10 mm)	6.0 ± 5.2	5.2 ± 2.6	< 0.01
ESR, mm/h	48.8 ± 29.2	41.3 ± 27.2	< 0.01
CRP, mg/dL	2.6 ± 3.3	1.7 ± 2.3	< 0.01
DAS28-ESR score	5.6 ± 1.4	4.7 ± 1.5	< 0.01
DAS28-CRP score	4.9 ± 1.4	4.0 ± 1.5	< 0.01
SDAI score	30.3 ± 14.3	21.3 ± 12.8	< 0.01
CDAI score	27.8 ± 13.2	19.7 ± 11.9	< 0.01
RAPID3 score	15.9 ± 6.0	12.4 ± 6.6	< 0.01
The proportion of patients with remission			
DAS28-ESR, *n* (%)	21 (3.5)	153 (10.7)	< 0.01
DAS28-CRP, *n* (%)	38 (6.4)	305 (21.4)	< 0.01
SDAI, *n* (%)	8 (1.3)	112 (7.7)	< 0.01
CDAI, *n* (%)	3 (0.5)	69 (4.8)	< 0.01
RAPID3, *n* (%)	12 (2.0)	128 (8.9)	< 0.01
Boolean-based criteria, *n* (%)	5 (0.8)	82 (5.8)	< 0.01

### Distribution of FAA

Among patients with FAA, ankle was the most common tender (*n* = 427/598, 71.4%) or swollen (*n* = 356/598, 59.5%) joint. Of MTP joints, the third MTP joint (*n* = 185/598, 30.9%) was the most common tender joint, followed by the second (*n* = 177, 29.6%), fourth (*n* = 165, 27.6%), first (*n* = 157, 26.3%), and fifth (*n* = 113, 18.9%) MTP joints. The third MTP joint (*n* = 134/598, 22.4%) was the most common swollen joint, followed by the second (*n* = 128, 21.4%), fourth (*n* = 110, 18.4%), first (*n* = 89, 14.9%), and fifth (*n* = 63, 10.5%) MTP joints.

### High disease activity and increased radiographic damage in patients with FAA

**Fig. 1:**
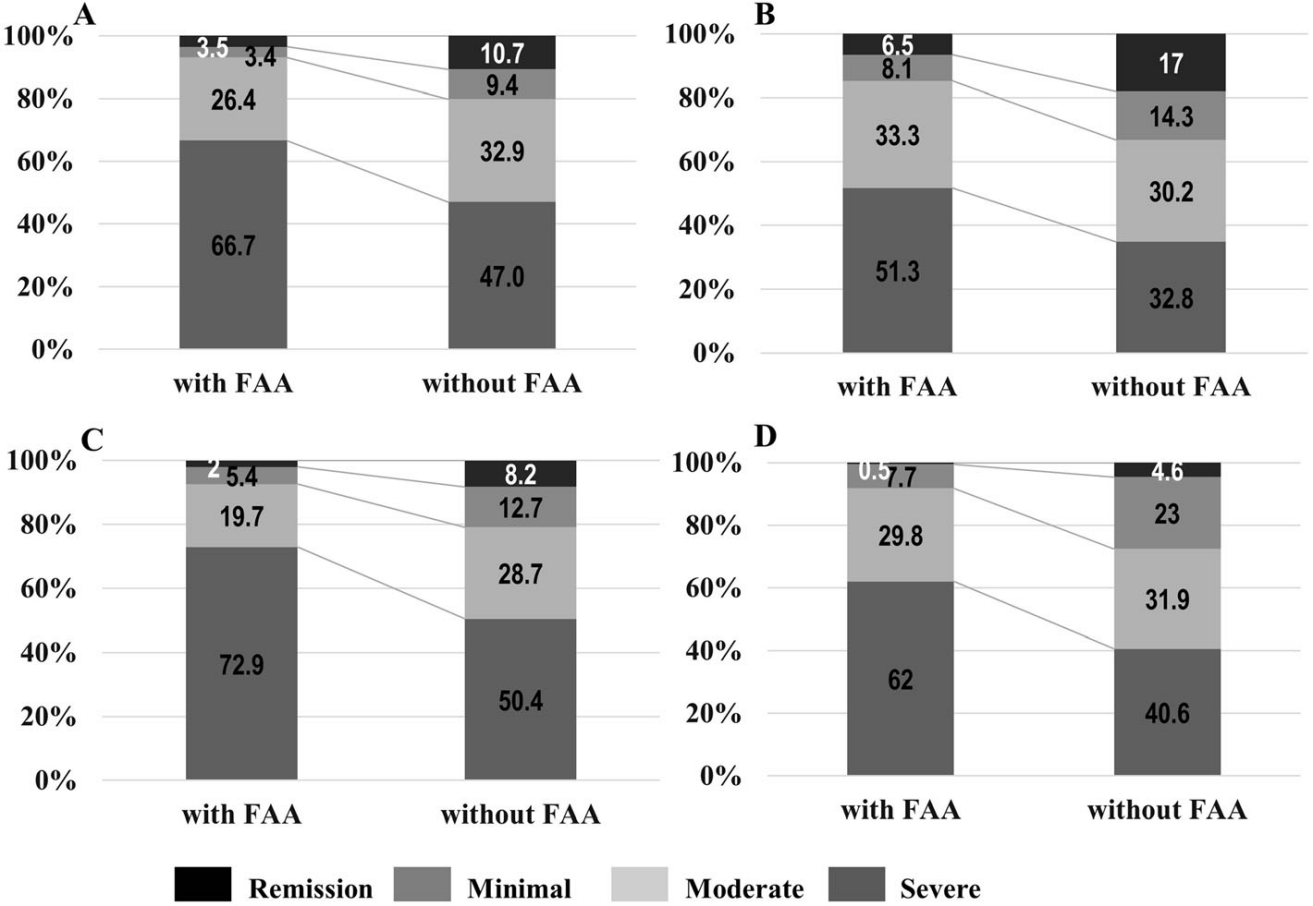
The proportion of patients with and without FAA according to each disease activity index. **a** DAS28-ESR, **b** SDAI, **c** CDAI, **d** RAPID3: Patients with FAA were worse disease activity than those without FAA as per DAS28, SDAI, CDAI, and RAPID3. Among various indices, patients with FAA were less likely to achieve remission in SDAI (*n* = 6, 1.0%) and CDAI (*n* = 3, 0.5%) than in DAS28-ESR (*n* = 21, 3.5%), DAS28-CRP (*n* = 38, 6.4%), RAPID3 (*n* = 12, 2.0%). CDAI, clinical disease activity index; DAS, disease activity score; ESR, erythrocyte segmentation rate; SDAI, simplified disease activity index; RAPID3, routine assessment of patient index data 3

The 44 SJCs, 44 TJCs, PGA, and EGA showed higher scores in patients with FAA than in those without FAA (Table 1). Of the total number of patients, the remission rate was 8.6% (*n* = 174), 17.0% (*n* = 343), 5.9% (*n* = 120), 3.5% (*n* = 72), 6.9% (*n* = 140), and 4.3% (*n* = 87) as per DAS28-ESR, DAS28-CRP, SDAI, CDAI, RAPID3 and Boolean-based definition of remission, respectively. Patients with FAA had higher PGA, EGA, DAS28, SDAI, CDAI, and RAPID3 scores (Table 1). Patients with FAA were less likely to achieve remission in SDAI (*n* = 6, 1.0%) and CDAI (*n* = 3, 0.5%) than in DAS28-ESR (*n* = 21, 3.5%), DAS28-CRP (*n* = 38, 6.4%), RAPID3 (*n* = 12, 2.0%) (Fig. 1). Five patients (0.8%) with FAA achieved remission as per the Boolean-based definition of remission.

The radiographic results were available for a total of 1737 hands and 1297 ft. Patients with FAA showed more bone erosion not only on radiographs of the foot [total *n* = 1265, 36.7% (*n* = 153) for patients with FAA vs 29.1% (*n* = 247) for those without FAA, *p* = 0.007] but also on radiographs of the hand [total *n* = 1693, 40.0% (*n* = 197) for patients with FAA vs 34.8% (*n* = 417) for those without FAA, *p* = 0.05] (Table 1).

On multivariate analysis for index-based remission, the presence of FAA was significantly associated with a reduced likelihood of remission in DAS28 ESR, DAS28-CRP, SDAI, CDAI, and RAPID3 after adjusting for age, duration of disease, presence of RF, presence of anti-CCP, existence of lung disease, use of MTX, and previous use of bDMARDs (Table 2).

**Table 2:** Multivariate analysis^a^ for no remission according to each clinical index

	OR	95% CI	*P*-value
DAS28-ESR, no remission			
Disease duration, years	1.02	1.00–1.05	0.10
Positive for rheumatoid factor	2.18	1.47–3.22	< 0.00
Previous use of bDMARDs	4.37	2.00–9.54	< 0.00
Presence of FAA	3.43	2.01–5.84	< 0.00
DAS28-CRP, no remission			
Presence of lung disease	3.53	1.08–11.53	0.04
Positive for rheumatoid factor	1.64	1.18–2.29	< 0.00
Previous use of bDMARDs	4.25	2.47–7.31	< 0.00
Presence of FAA	3.59	2.43–5.33	< 0.00
SDAI, no remission			
Previous use of bDMARDs	6.77	2.13–21.5	< 0.00
Presence of FAA	6.33	2.75–14.6	< 0.00
CDAI, no remission			
Previous use of bDMARDs	5.94	1.44–24.49	< 0.00
Presence of FAA	7.59	2.37–24.33	< 0.00
RAPID, no remission			
Use of MTX	2.6	0.94–7.23	0.07
Previous use of bDMARDs	8.22	2.59–26.11	< 0.00
Presence of FAA	5.57	2.69–11.52	< 0.00

^a^Factors known to be associated with remission such as age, disease duration, the positivity of RF, positivity of anti-cyclic citrullinated peptide, the existence of lung disease, use of MTX, previous use of bDMARDs were included for multivariate logistic regression analysis

### Near misses by Boolean-based definition of remission caused by PGA, which was not affected by FAA

In this study, 38.2% (*n* = 766), 31.1% (*n* = 623), 12.5% (*n* = 250), 13.9% (*n* = 279), and 4.3% (*n* = 87) of the patients satisfied 0, 1, 2, 3, and 4 items of the four items included in the Boolean-based definition of remission, respectively. The most common reason for the near misses of Boolean-based definition of remission (i.e., patients satisfying only 3 items) was the PGA score > 1 (*n* = 261, 93.5%), followed by CRP level of > 1 mg/dL (*n* = 7, 2.5%), SJC > 1 of SJC (*n* = 7, 2.5%), and TJC > 1 of TJC (*n* = 4, 1.4%). Among patients with near misses of Boolean-based definition of remission due to PGA, only 14.2% (*n* = 37) patients had FAA.

## Discussion

KOBIO registry was established to investigate the effects and adverse events of bDMARDs. In Korea, since July 2009, the government has provided expanded benefit coverage in the national health insurance for patients with rare incurable diseases (the Exempted Calculation of Health Insurance for rare, incurable diseases). However, only seropositive RA patients (positive either for RF or anti-CCP antibody) have this advantage. Therefore, presence of RF and anti-CCP antibody was high in this cohort. Moreover, because most of the enrolled patients were considered to initiate or change bDMARD treatment, the proportion of patients with RA-associated lung diseases was low.

In the current study, the prevalence of FAA (29.2%) was far less than that reported in a previous study, in which 70% of the patients among the cohort with early RA with a symptom duration of < 3 years had at least one or more MTP joint pain and swelling [14]. However, in that study, these cases were classified as RA according to the 1987 ACR criteria known to detect relatively advanced RA, while most patients in the current study were diagnosed as RA using the 2010 ACR classification criteria, which is known to detect early RA. Accordingly, patients with RA diagnosed based on 1987 ACR criteria may showhave radiographic damages, even those with < 3 years since the onset of symptoms.

There are several factors for FAA other than RA, including high BMI, occupation, or other concurrent medical conditions, such as gout or osteoarthritis (OA). These factors might affect the result of the present study, but their effect may be not influential enough to mask the burden of RA on FAA. Although increased BMI is associated with arthritis in the lower extremities [15, 16], the BMI between patients with and without FAA in the current cohort was not statistically different, and the mean of the BMI was 22.6 ± 3.3 kg/m^2^, which is far less than that among patients in those studies (32.1 ± 8.4 kg/m^2^) [16]. The coexistence of gout and RA has been rarely reported [17]. About 40% of patients with RA have concurrent foot OA [18], and the most commonly affected joint in foot OA is the first MTP joint, and OA is rarely associated with ankle joints; in the current study, the most commonly affected joints in FAA were ankle joints, followed by the third and second MTP joints. Further, the treating physician assessed the joints and considered them as swollen or tender joints only if they were considered to be associated with RA in the present study.

Presence of FAA denotes more aggressive and severe disease status. Predominant foot progressors showed more radiographic progression for the same changes in DAS28 than hand and foot progressors, predominantly hand progressors, or non-progressors [19, 20]. In the current study, patients with FAA showed higher overall activity than those without FAA, and noticeable radiographic damage was more frequent not only in feet but also in hands (Table 1). Presence of FAA is a consistently significant factor for no remission in all disease activity index (Table 2). Notably, patients with FAA of longer duration are less likely to be in remission. (Additional file 1: Table S2).

There are still controversies regarding the 28 joint count-based indices, which represent entire joints, except foot and ankle joints. Although 29–30% of patients with DAS28 remission have foot arthritis, the remission rate and joint activity over time were comparable between the 28-joint count-based indices and 32-joint count-based indices [10, 21]. The basis of representativeness of those indexes is that the presence of FAA may be implicated in subjective measures, such as PGA and EGA, albeit they do not include FAA in the 28-joint count. In the current study, among patients with DAS28 remission, PGA was significantly higher among patients with FAA than among those without FAA (Additional file 1: Table S1). Of note, PGA may be affected by other conditions, such as depression or fibromyalgia. Unsatisfied PGA is the limiting factor for Boolean-based complete remission [20], and factors other than RA such as depression and fibromyalgia associated with failure to meet PGA ≤ 1 [22]. In the current study, the most common reason for the near-miss of Boolean-based complete remission was also unsatisfied PGA, but only 14.2% of these patients had FAA. Therefore, although subjective components of 28-joint count-based index compensate for missed FAA, PGA alone is not enough and both PGA and EGA should be considered. Among patients with remission as per SDAI and CDAI, which involves EGA and is more weighted on PGA than DAS28, only 6.7% (8/120, SDAI remission) and 4.2% (3/72, CDAI remission) patients were diagnosed with FAA, whereas 12.1% (21/174) and 11.1% (38/343) patients with remission as per DAS28-ESR and DAS28-CRP had FAA, respectively, in the present study. Accordingly, among the 28-joint count-based index, SDAI and CDAI may be more applicable to reflect actual disease activity concerning FAA.

The current study has several limitations. First, assessments were conducted every year so we cannot be sure of the status during the 12 months. However, despite this limitation, this is one of the largest RA cohorts worldwide. Second, we defined FAA as the presence of swollen or tender joints in the first to fifth MTP or ankle joints, which may provoke controversy because arthritis included both swollen and tender joint in most studies. Prevalence of FAA was 19.8% (*n* = 403) in the present study cohort if FAA includes both swollen and tender joints, which is far less than that in previous studies as discussed above. Because it is challenging to assess swollen joint count on occasion [23], and underestimation rather than overestimation is suspected in this large cohort, we considered to define FAA as one or more tender or swollen joints in the ankle and/or first to fifth metatarsophalangeal (MTP) joints. Further studies using ultrasound-detected synovitis may be needed, but this needs intense effort to evaluate more than 2000 patients as in the present study, and there may be issues in standardizing the ultrasound technique as it is may vary with the examiner.

## Conclusion

In conclusion, approximately 30% of patients with RA have FAA. Presence of FAA represents a more severe disease activity status and is an independent risk factor for non-remission in patients with RA. Among clinical indices, SDAI and CDAI best represent FAA.

## Additional files

Additional file 1: **Table S1.** Demographic and clinical data of patients with DAS28-ESR remission (total n=174). (DOCX 25 kb)

Additional file 2: Supplementary file. (DOCX 14 kb)

## Data Availability

The datasets used and/or analyzed during the current study are available from the corresponding author on reasonable request. Korean College of Rheumatology Biologics &Targeted therapy Registry web site: http://www.kobio.or.kr/kobio/

## Abbreviations

ACR/EULAR: American College of Rheumatology/European League Against Rheumatism
anti-CCP: anti-cyclic citrullinated peptide
bDMARDs: biologic disease-modifying anti-rheumatic drugs
BMI: Body mass index
CDAI: clinical disease activity index
CRP: C-reactive protein
DAS: Disease activity score
EGA: Evaluator’s global assessment
ESR: Erythrocyte sedimentation rate
FAA: Feet and/or ankle arthritis
KOBIO: The Korean nationwide BIOlogics registry
MTP: Metatarsophalangeal
MTX: Methotrexate
OA: Osteoarthritis
PGA: Patient’s global assessment
RA: Rheumatoid arthritis
RAPID3: Routine assessment of patient index data 3
RF: Rheumatoid factor
SDAI: Simplified disease activity index
SJCs: Swollen joint counts
TJCs: Tender joint counts
