# Supplementary material for: Prevalence of feet and ankle arthritis and their impact on clinical indices in patients with rheumatoid arthritis: a cross-sectional study
Source: BMC Musculoskelet Disord. 2019 Sep 11;20:420. doi: 10.1186/s12891-019-2773-z (PMC6737695; doi:10.1186/s12891-019-2773-z)
Supplement: Supplementary file 1 — Table S1. Demographic and clinical data of patients with DAS28-ESR remission (total n=174). (DOCX 25 kb) [file 12891_2019_2773_MOESM1_ESM.docx]

**Table S1**. Demographic and clinical data of patients with DAS28-ESR remission (total n=174).

|  | With FAA | Without FAA | P-values |
| --- | --- | --- | --- |
|  | (21, 12.1%) | (153, 87.9%) |  |
| **Demographic Characteristics** |  |  |  |
| Female, n (%) | 17 (81.0) | 126 (82.4) | 1.00 |
| Age at the time of enrollment, years | 50.7 ± 9.4 | 53.5 ± 11.9 | 0.30 |
| Disease duration, years | 4.8 ± 5.0 | 6.4 ± 6.3 | 0.26 |
| Body mass index, kg/m^2^ | 22.2 ± 2.5 | 22.8 ± 3.0 | 0.44 |
| Current/ex-smoker, n (%) | 6 (28.6%) | 26 (17.0%) | 0.23 |
| Presence of RA-associated lung diseases^*^, n (%) | 0 (0%) | 2 (1.3%) | NA |
| Positive for rheumatoid factor, n (%) | 12 (75.0) | 111 (77.6) | 0.76 |
| Positive for anti-cyclic citrullinated peptide, n (%) | 17 (81.0) | 109 (72.7) | 0.60 |
| **Radiographic damage** |  |  |  |
| Hands X-ray ^¶^ |  |  |  |
| Erosion, n (%) | 6 (33.3) | 26 (21.5) | 0.37 |
| Joint space narrowing, n (%) | 6 (33.3) | 37 (30.6) | 1.00 |
| Feet X-ray ^ǂ^ |  |  |  |
| Erosion, n (%) | 3 (17.6) | 23 (26.1) | 0.55 |
| Joint space narrowing, n (%) | 3 (17.6) | 16 (18.2) | 1.00 |
| **Disease activity** |  |  |  |
| Swollen joint count (44 joints examined) | 2.6 ± 4.5 | 0.2 ± 0.5 | 0.02 |
| Tender joint count (44 joints examined) | 2.0 ± 2.2 | 0.3 ± 0.6 | <0.00 |
| Patients Global Assessment (1-10 mm) | 3.5 ± 2.5 | 2.2 ± 1.7 | 0.03 |
| Evaluator's Global Assessment (1-10 mm) | 2.4 ± 1.7 | 1.7 ± 1.4 | 0.09 |
| ESR, mm/hr | 7.5 ± 5.8 | 10.4 ± 7.1 | 0.07 |
| CRP, mg/dL | 0.4 ± 0.9 | 0.6 ± 1.6 | 0.56 |
| DAS28-ESR score | 2.0 ± 0.4 | 1.9 ± 0.5 | 0.24 |
| DAS28-CRP score | 2.1 ± 0.6 | 1.7 ± 0.5 | <0.00 |
| SDAI score | 8.1 ± 5.4 | 4.5 ± 3.2 | <0.00 |
| CDAI score | 7.9 ± 5.4 | 4.4 ± 3.2 | <0.00 |
| RAPID3 score | 7.1 ± 5.0 | 5.1 ± 3.9 | 0.03 |
| **Medication** |  |  |  |
| Current glucocorticoid use, n (%) | 13 (65.0) | 94 (61.4) | 0.81 |
| Daily dose (prednisolone equivalent), mg | 2.4 ± 2.2 | 2.7 ± 4.3 | 0.80 |
| Current use of MTX, n (%) | 19 (90.5) | 142 (92.8) | 0.66 |
| Previous use of bDMARDs, n (%) | 1 (4.8) | 8 (5.2) | 1.00 |

^*^ include pleuritis, interstitial lung disease, bronchiolitis obliterans organizing pneumonia, and obliterative bronchiolitis.

^¶^Data of hands x-ray were available in 139 patients.

^ǂ^ Data of feet x-ray were available in 105 patients.

**Table S2**. Demographic and clinical data of DAS28-ESR remission patients with foot and/or ankle arthritis (total n= 595).

|  | | DAS28-ESR Remission | | DAS28-ESR Non-remission | | P-values | |
| --- | --- | --- | --- | --- | --- | --- | --- |
|  |  | (21, 3.5%) | | (574, 96.5%) | |  |  |
| **Demographic Characteristics** | |  | |  | |  | |
| Female, n (%) | | 17 (87.0) | | 500 (87.1) | | 0.50 | |
| Age at the time of enrollment, years | | 50.7 ± 9.4 | | 54.3 ± 12.6 | | 0.20 | |
| Disease duration, years | |  | |  | |  | |
| Disease duration < 1 year, n (%) | | 4 (19.0) | | 95 (16.6) | | 0.77 | |
| Disease duration < 2 years, n (%) | | 10 (47.6) | | 159 (27.8) | | 0.05 | |
| Disease duration < 3 years, n (%) | | 10 (47.6) | | 189 (33.0) | | 0.17 | |
| Disease duration < 4 years, n (%) | | 11 (52.4) | | 228 (39.9) | | 0.25 | |
| Disease duration < 5 years, n (%) | | 14 (66.7) | | 257 (44.9) | | 0.05 | |
| Disease duration ≥ 5 years, n (%) | | 7 (33.3) | | 315 (55.1) | | 0.05 | |
| Body mass index, kg/m^2^ | | 22.2 ± 2.4 | | 22.6 ± 3.6 | | 0.67 | |
| Current/ex-smoker, n (%) | | 6 (28.6) | | 83 (14.5) | | 0.11 | |
| Presence of RA-associated lung diseases, n (%)^*^ | | 0 | | 21 (3.7) | | NA | |
| Positive for rheumatoid factor, n (%) | | 15 (71.4) | | 475 (85.7) | | 0.12 | |
| Positive for anti-cyclic citrullinated peptide, n (%) | | 12 (75.0) | | 390 (85.0) | | 0.29 | |
| **Radiographic damage** | |  | |  | |  | |
| Hand X-ray | |  | |  | |  | |
| Erosion, n (%) | | 6 (33.3) | | 246 (52.1) | | 0.63 | |
| Joint space narrowing, n (%) | | 6 (33.3) | | 219 (46.3) | | 0.34 | |
| Feet X-ray | |  | |  | |  | |
| Erosion, n (%) | | 4 (23.5) | | 169 (42.7) | | 0.12 | |
| Joint space narrowing, n (%) | | 3 (17.6) | | 104(26.1) | | 0.58 | |
| **Medication** | |  | |  | |  | |
| Current glucocorticoid use, n (%) | | 13 (61.9) | | 482 (84.0) | | 0.02 | |
| Daily dose (prednisolone equivalent), mg | | 0.71 ± 0.52 | | 0.89 ± 0.41 | | 0.12 | |
| Current use of MTX, n (%) | | 19 (90.5) | | 546 (95.1) | | 0.29 | |
| Previous use of bDMARDs, n (%) | | 1(4.8) | | 143 (24.9) | | 0.04 | |
| **Disease activity** | |  | |  | |  | |
| Swollen joint count (44 joints examined) | | 2.6 ± 4.5 | | 9.2 ± 6.8 | | < 0.02 | |
| Tender joint count (44 joints examined) | | 2.0 ± 2.2 | | 12.3 ± 8.6 | | < 0.02 | |
| Patients Global Assessment score (1-10 mm) | | 3.5 ± 2.5 | | 6.8 ± 2.2 | | < 0.02 | |
| Evaluator's Global Assessment score (1-10 mm) | | 2.4 ± 1.7 | | 6.1 ± 2.0 | | < 0.02 | |
| ESR, mm/h | | 7.5 ± 5.8 | | 50.6 ± 28.4 | | < 0.02 | |
| CRP, mg/dL | | 0.4 ± 0.9 | | 2.6 ± 3.3 | | < 0.02 | |
| DAS28-ESR score | | 2.0 ± 0.4 | | 5.7 ± 1.3 | | < 0.02 | |
| DAS28-CRP score | | 2.1 ± 0.6 | | 5.1 ± 1.3 | | < 0.02 | |
| SDAI score | | 8.1 ± 5.4 | | 31.1 ± 13.9 | | < 0.02 | |
| CDAI score | | 7.9 ± 5.4 | | 28.6 ± 12.8 | | < 0.02 | |
| RAPID3 score | | 7.0 ± 4.9 | | 16.3 ± 5.8 | | < 0.02 | |
| **The proportion of patients with remission** | |  | |  | |  | |
| DAS28-CRP, n (%) | | 16 (76.2) | | 22 (3.8) | | < 0.02 | |
| SDAI, n (%) | | 4 (19.0) | | 3 (0.5) | | < 0.02 | |
| CDAI, n (%) | | 2 (9.5) | | 1 (0.2) | | < 0.02 | |
| RAPID3, n (%) | | 3 (14.3) | | 9 (1.6) | | < 0.02 | |
| Boolean-based criteria, n (%) | | 2 (9.5) | | 0 (0) | | < 0.02 | |

^*^include pleuritis, interstitial lung disease, bronchiolitis obliterans organizing pneumonia, and obliterative bronchiolitis.
