# Supplementary material for: Prevalence of feet and ankle arthritis and their impact on clinical indices in patients with rheumatoid arthritis: a cross-sectional study
Source: BMC Musculoskelet Disord. 2019 Sep 11;20:420. doi: 10.1186/s12891-019-2773-z (PMC6737695; doi:10.1186/s12891-019-2773-z)
Supplement: Supplementary file 2 — Supplementary file. (DOCX 14 kb) [file 12891_2019_2773_MOESM2_ESM.docx]

Supplementary.

This study was approved by the institutional review boards of Seoul National University Hospital, Hanyang University, Catholic University of Korea Seoul St. Mary’s Hospital, Chonnam National University Hospital, Samsung Medical Center, Wonkwang University Hospital, Asan Medical Center, Kyunghee University Medical Center, Eulji General Hospital, Ajou University Hospital, Gyeongsang National University Hospital, Daegu Catholic University Medical Center, Hallym University Kangnam Sacred Heart Hospital, Korea University Ansan Hospital, Inha University Hospital, Anynang SAM Medical Center, Gachon University Gil Medical Center, Jeju National University Hospital, Konyang University Hospital, Chungnam National University Hospital, Catholic University of Korea Incheon St Mary’s Hospital, Korea University Guro Hospital, Dong-A University Hospital, Daegu Catholic University Medical Center, Severance Hospital, Soonchunhyang University Bucheon Hospital, Konkuk University Medical Center, Kyungpook National University, Chung-Ang University Hosptial, Ewha woman’s University Hospital, Gangneung Asan Hospital, SMG-SNU Boramae Medical Center, Kosin University Gospel Hospital, Bundang Jesaeng Hospital, Soonchunhyang University Seoul Hospital, Kyunghee University Hospital at Gangdone, National Police Hospital, and Soonchunhyang University Cheonan Hospital (all 38 participating institutions).
